# Supplementary figures and images for: Exploring the classification of cancer cell lines from multiple omic views
Source: PeerJ. 2020 Aug 18;8:e9440. doi: 10.7717/peerj.9440 (PMC7441922; doi:10.7717/peerj.9440)

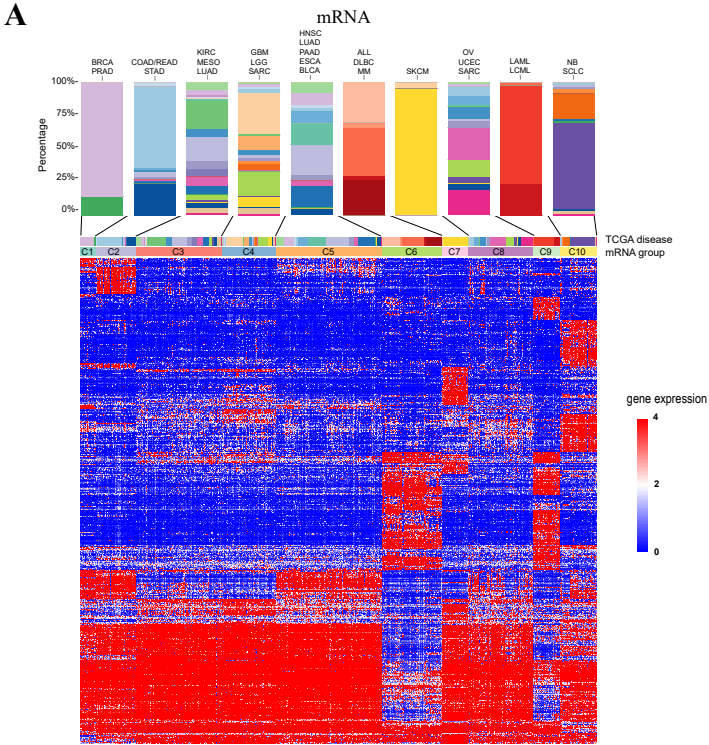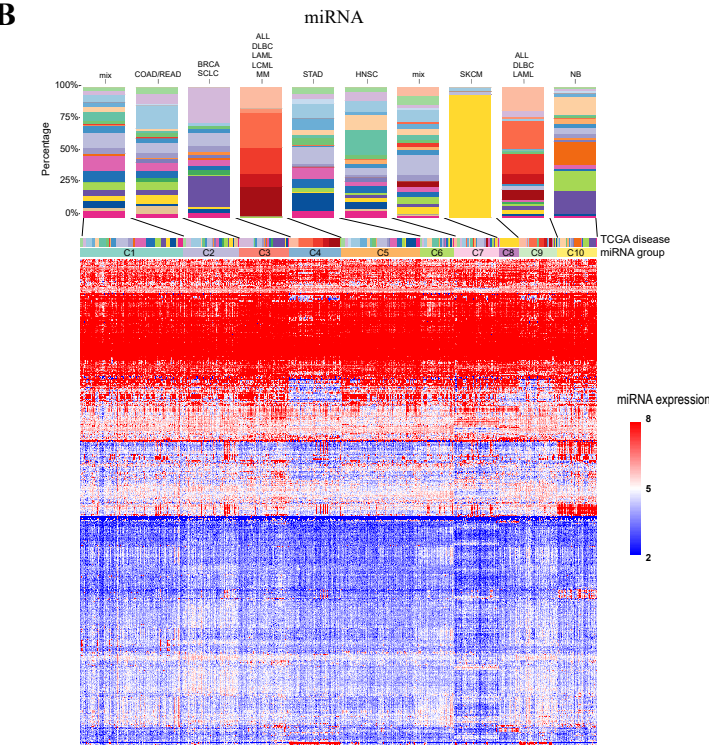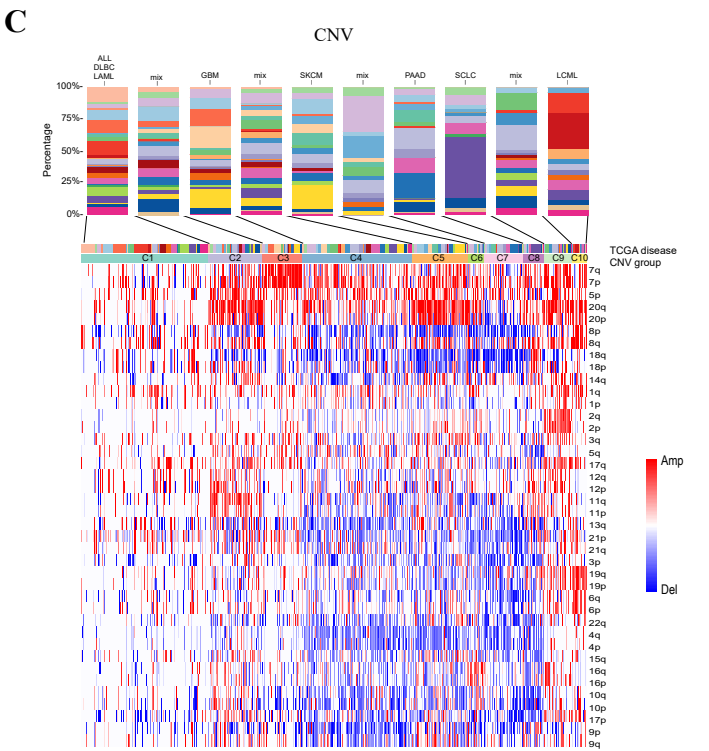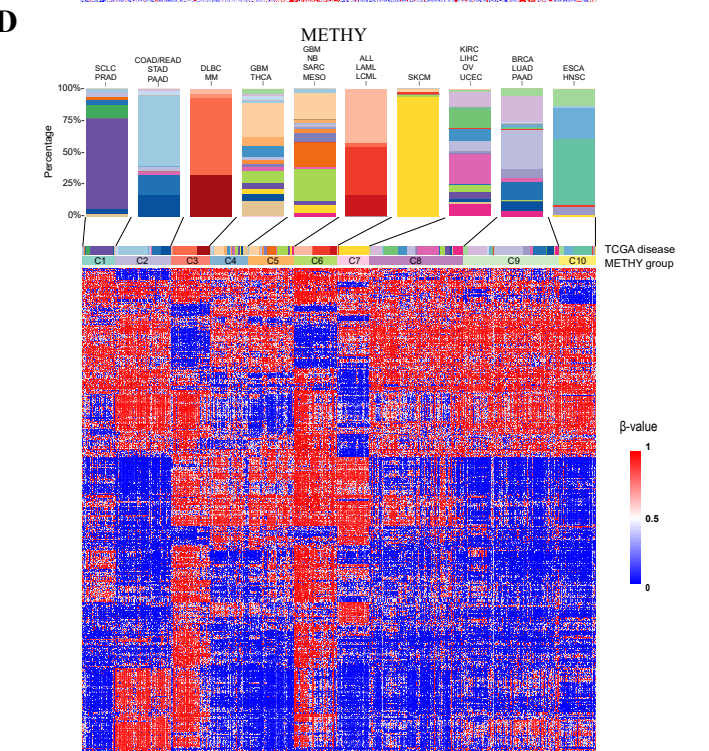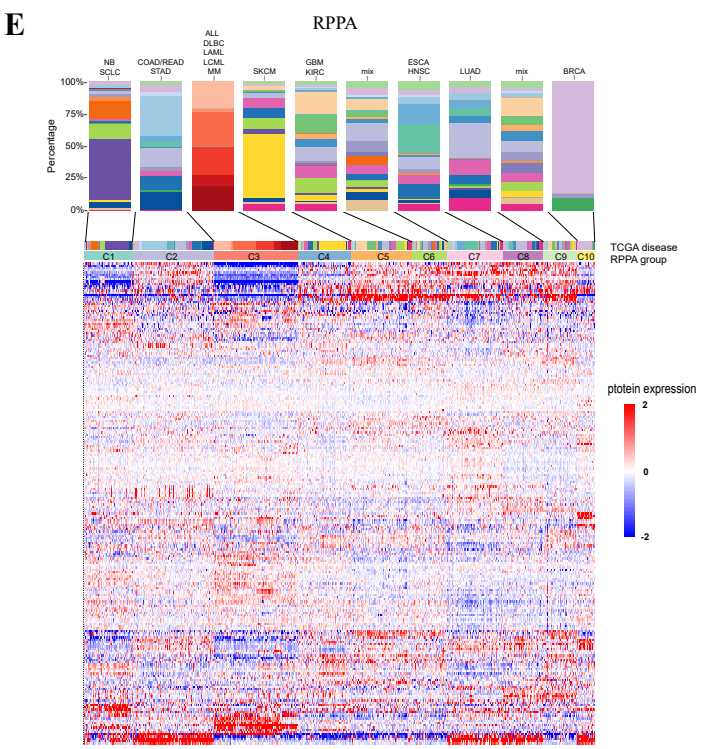

TCGA disease abbreviation

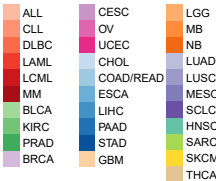

Supplement: Figure S1 — Hierarchical clustering of (A) mRNA, (B) miRNA, (C) CNV, (D) METHY and (E) RPPA data. Types of cancer cell line are color-coded as shown in the right corner. The first track represents cell lines of TCGA disease. The second track represents the single omics clustering group. A bar graph was used to show cancer types and the proportion of cell lines in each cluster. The dominant cancer types were marked on the top of the bar graph. [file peerj-08-9440-s001.pdf]

C

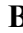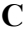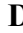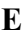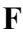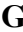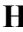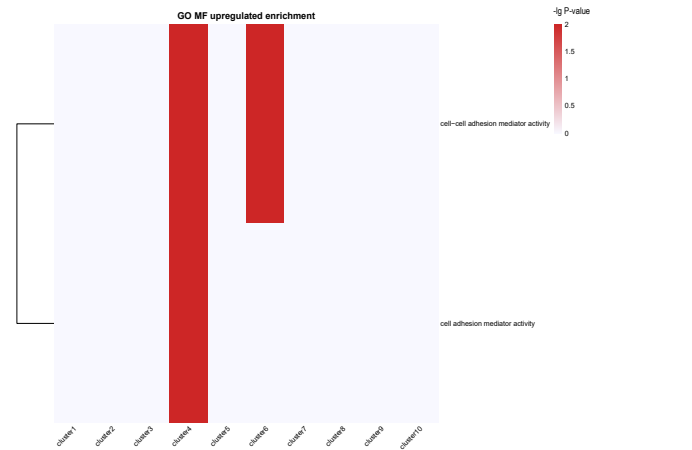

Supplement: Figure S2 — (A) KEGG enrichment heatmap of down-regulated genes. (B) KEGG enrichment heatmap of up-regulated genes. (C) GO biological process enrichment heatmap of down-regulated genes. (D) GO biological process enrichment heatmap of up-regulated genes. (E) GO cellular component enrichment heatmap of down-regulated genes. (F) GO cellular component enrichment heatmap of up-regulated genes. (G) GO molecular function enrichment heatmap of down-regulated genes. (H) GO molecular function enrichment heatmap of up-regulated genes. Deeper red color signifies greater enrichment score in all panels. [file peerj-08-9440-s002.pdf]

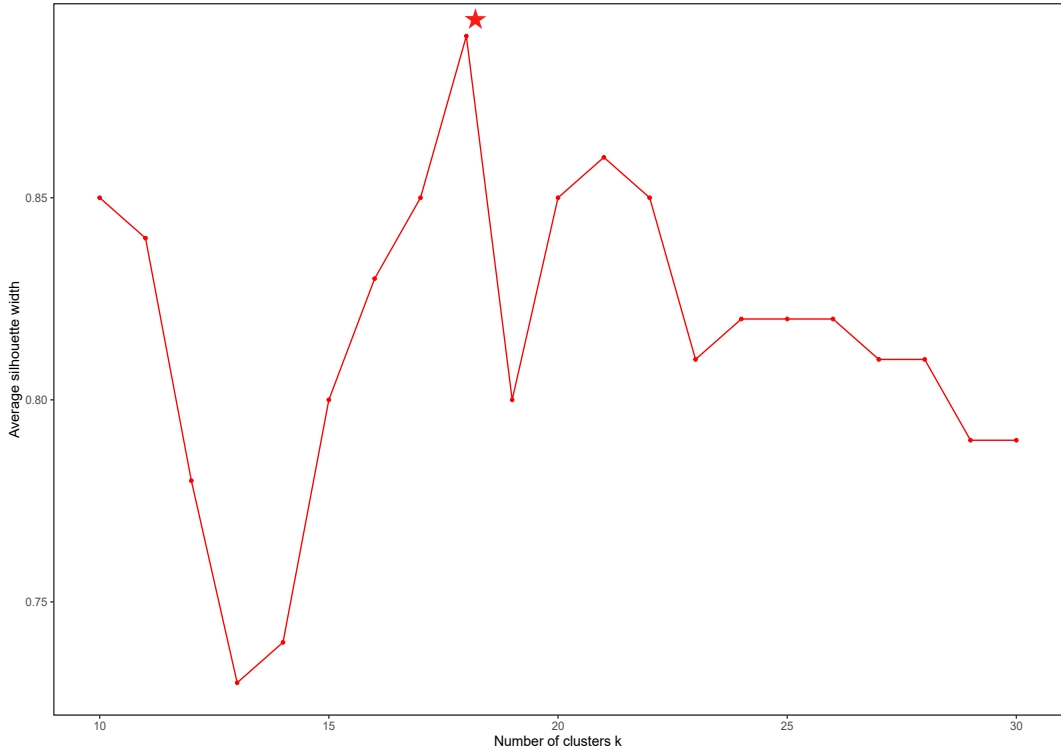

Supplement: Figure S3 [file peerj-08-9440-s003.pdf]

Dominant cancer types of clusters in integrated multiple omics clustering

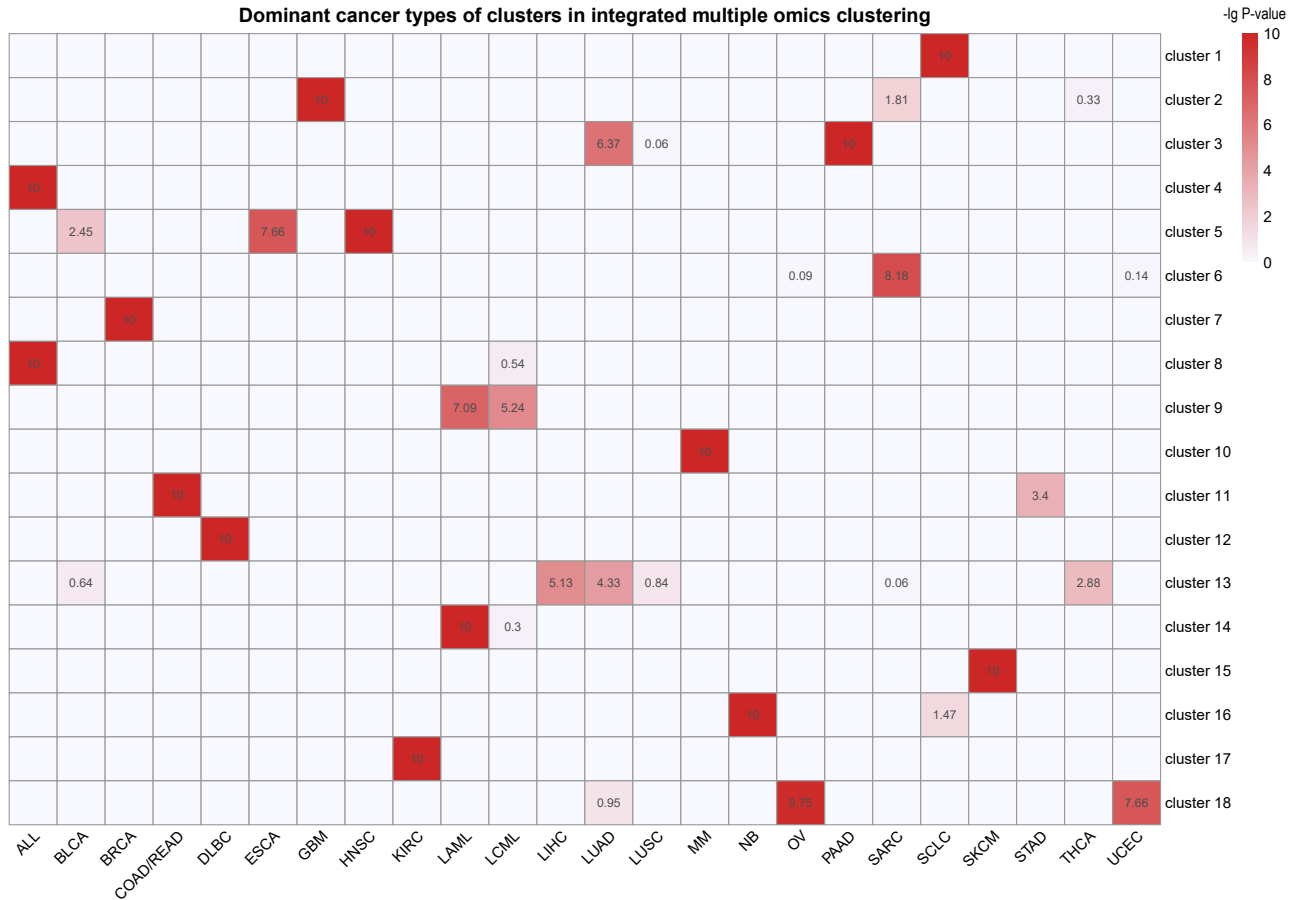

Supplement: Figure S4 — A hypergeometric distribution was used to calculate the P-value for cancer types in each cluster. The rows represent clusters, and the columns represent cancer types. The values represent the –lg(P-value) of cancer types. Cancer types with –lg(P-value) > 3 in each cluster were defined as dominant cancer types. All the blank cells mean the instances of P-value = 0. [file peerj-08-9440-s004.pdf]

**A****Paclitaxel**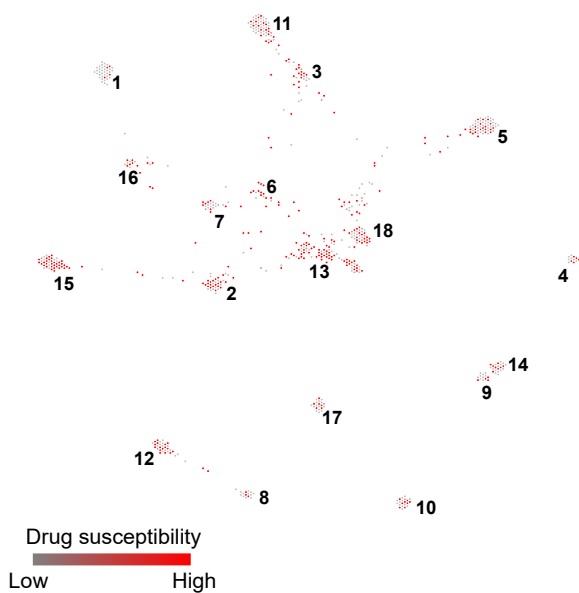**B****L-685458**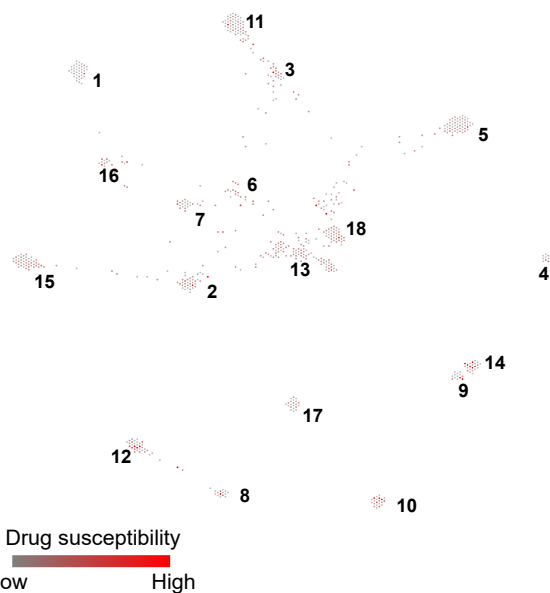**C****PLX4720**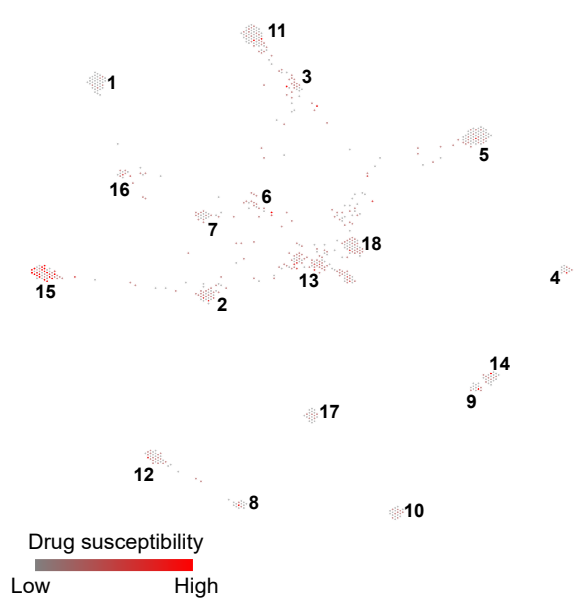**D****RAF265**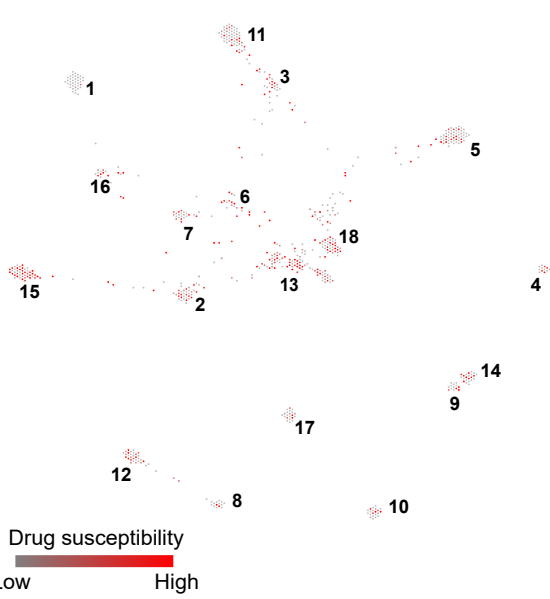

Supplement: Figure S5 — The TumorMap layout was as described for Figure 4. Drug susceptibility for (A) Paclitaxel, (B)L-685458, (C) PLX4720 and (D) RAF265. Increasing red colors indicate increasing sensitive degree. [file peerj-08-9440-s005.pdf]
